# Boldness is not associated with dynamic performance capacity in hermit crabs

**DOI:** 10.1098/rsbl.2023.0224

**Published:** 2023-07-26

**Authors:** Winne Courtene-Jones, Mark Briffa

**Affiliations:** School of Biological and Marine Sciences, University of Plymouth, Drake Circus, Plymouth PL4 8AA, UK

**Keywords:** animal personality, dynamic performance, boldness, hermit crab

## Abstract

Boldness, the way an individual reacts to risk, is a commonly studied personality trait in animals. Consistent among-individual differences in startle response durations (latency to recover from a startling stimulus) are frequently assumed to reflect variation in boldness. An alternative explanation is that these latencies are not directly driven by variation in responses to information on risk, but by underlying differences in dynamic performance capacities. Here we investigate this possibility by analysing relationships between locomotory speed, a measure of whole-body dynamic performance capacity in hermit crabs, and startle response duration, a repeatable latency measure used as an index of boldness. Individuals differed in mean startle response duration, in the consistency of their startle responses, in their reaction norms across repeated observations, and mean startle responses increased with crab mass. However, there were no relationships between startle responses and locomotory speed. This indicates that startle responses do not reflect underlying performance capacities and suggests that they provide insight into differences in how individuals respond to risky situations. Since similar latencies are used as measures of boldness in other animals, we suggest that potential relationships between apparent boldness and performance capacity should be tested.

## 1. Introduction

Animal personality in its narrowest sense refers to the presence of significant differences among individuals in their behavioural means derived from repeated observations, typically quantified by repeatability and its confidence intervals. Formally, repeatability, is the proportion of variance owing to differences between individuals (between individual variance; *V*_BA_) out of the total variance including that within individuals (*V*_WA_), the latter also called unpredictability [1], predictability [2], residual [3] or intra-individual behavioural variance [4]. Within-individual variance itself can differ among individuals and thus can be considered another aspect of animal personality, along with differences between individuals in how their behaviour changes plastically over time or across gradients, where individuals show different behavioural reaction norms [5]. Demonstrating the presence of narrow sense animal personality does not in itself provide insight into its underlying proximate or evolutionary causes [6] but the terms used to describe behaviours, once demonstrated as repeatable, can carry connotations of particular causal factors [7]. For instance, of the five major axes of animal personality, as adapted from human psychology [8], ‘boldness' has received particular attention. In lay-terms boldness equates to ‘confidence' and biologically it has been defined as ‘an individual's reaction to any risky situation' [8, p. 295]. Thus boldness, as generally understood, implies differences in how individuals react to information on risk, i.e. their ‘risk-coping strategy'. When confronted with a potentially dangerous event, animals may either flee or hide, which includes adopting a protective posture. In this latter case, bolder individuals re-emerge from hiding, and resume ongoing activity, more quickly compared with shyer individuals at the opposite end of a bold-shy continuum. An untested alternative to different risk-coping strategies is that among-individual differences in apparent boldness instead primarily result from variation in dynamic performance capacities, defined as the ability to perform sustained and rapid movement [9]. The latter could be indirectly compatible with differences in risk-coping, if boldness covaries with performance capacities as suggested by the pace of life syndrome hypothesis (POLS) [7,10] for example. However, risk-coping and performance capacities do not necessarily covary and performance capacities could directly drive consistent differences in apparent boldness if they constrain reaction times. Testing for a link between putative measures of boldness and dynamic performance is therefore a potentially important step in interpreting apparent boldness, particularly when measures used as indices of boldness are based on behavioural latencies that could be influenced by speed of movement.

A commonly used index of boldness is latency to recover from a startling stimulus, e.g. resumption of ongoing activities after a protective posture has been adopted [1,11] or re-emergence from a shelter [4,12,13]. European hermit crabs, *Pagurus bernhardus*, show both fleeing (from a visual cue [14]) and repeatable startle response durations when handled directly [12], measured as time spent tightly retracted into their empty gastropod shell, which hermit crabs use as portable shelters when threatened. This otherwise repeatable behaviour shows plasticity across gradients of risk, lasting longer [12] and being less predictable [2] in the presence of a predator. Collectively, these results are compatible with underlying differences in risk-coping strategies, but they do not rule out the possibility that they are driven (or driven in part) by underlying differences in performance capacity, particularly as startle responses correlate with metabolic rate [15,16]. Locomotion speed (measured as time taken to cover a set distance) has been validated an energetically significant activity [17] and a measure of dynamic performance capacity in a previous study on *P. bernhardus*, where slower moving individuals also performed energetically demanding ‘shell rapping' (which engages the same abdominal muscles used during startle responses) more slowly during subsequent agonistic contests [18]. While the role of abdominal musculature during locomotion remains to be fully elucidated it is likely that these muscles are engaged in order to adjust shell position, so as to maintain a posture where the shell will not interfere with locomotion [17]. Here we ask whether startle response durations are associated with this measure of dynamic performance capacity. If the speed of re-emergence is directly constrained by dynamic performance capacity, or if boldness covaries with performance as predicted by POLS, we should see a positive correlation between mean startle response duration and time taken to walk a set distance (i.e. slower walking individuals hide for longer). A negative correlation (i.e. faster moving individuals hide for longer) is unexpected if latency reflects dynamic performance but could be present for other reasons, for example if investment in high movement speed is part of a wider risk-avoidance syndrome. In this case we would also expect a negative correlation between movement speed and within individual variance in startle response duration, on the assumption that less predictable startle responses mitigate risk [2]. A lack of any correlation in either direction would indicate that startle response durations vary independently of dynamic performance and may instead differ across individuals for other reasons including differences in ability to detect or process information on risk, or differences in sensitivity to such information, i.e. differences in risk-coping strategy.

## 2. Methods

Hermit crabs were hand-collected from Hannafore Point, Cornwall UK between February–July 2013 (see the electronic supplementary material, S1). Following transport back to the laboratory in Plymouth, crabs were held in groups of approximately 100 individuals in constantly aerated and filtered seawater at 15°C to a depth of 30 cm, in a controlled 12 : 12 h light : dark environment, and fed ad libitum on small pieces of white fish. Prior to observation crabs were removed from their shells by carefully cracking the shell open using a bench vice, then sexed and weighed. Females were given new shells and returned to the sea and only males free of obvious injury or parasites were used in the experiment [19]. See the electronic supplementary material, S1 for further details. Male crabs were provided with a new *Littorina littorea* shell 50% of its optimal shell mass, calculated from a previous shell selection experiment, reduced shell size ensuring that continuous locomotion could be stimulated [18]. Males were then individually allocated to white plastic flat-bottomed dishes (20 cm diameter), filled with a 5 cm depth of aerated 15°C seawater. Startle responses were evoked by manually lifting a crab out of the seawater, causing it to retract into its shell [20], and replacing the shell, aperture upwards on the base of the dish. The duration of the response was timed using a stopwatch, until the crab re-emerged to the point where its second pair of walking legs contacted the substrate [12,20]. In 52 males eight startle responses were recorded per crab, over a period of 4 days, alternating between 16 and 5 h intervals between successive observations. Following this, each crab was placed into a clear plastic circular raceway (3.25 m in outer circumference, 6 cm wide raceway) filled to a depth of 5 cm with aerated 15°C seawater. Each crab was stimulated to walk by a series of light taps on its shell using a wooden probe. Once in motion the crab was followed by the probe at its own speed at a distance of one body length. If a crab stopped moving it immediately received an additional light tap, which caused walking to resume throughout the trial, and did not cause crabs to withdraw into their shell. We obtained two measures of locomotory performance: the time taken to cover 13 m (four laps), a measure of overall performance; and the duration of the fastest lap of the four, a measure of maximum exertion [18]. Owing to some data exclusions (see the electronic supplementary material, S2) a final sample size of 407 startle responses across 51 crabs was obtained.

### (a) Statistical methods

To determine whether variance in startle response durations differed among individuals we conducted an initial Levene's test. This revealed the presence of significant among individual differences in *V*_WI_ (see below), so prior to further analysis we log_10_ (1 + *x*) transformed the data, which yielded homogeneity of variance across individuals, and improved the normality of residuals in subsequent models. There were no correlations between either measure of dynamic performance capacity and mass (see the electronic supplementary material, S2), so we then ran a linear mixed effects model to determine the effects of crab mass, time taken to complete four laps, and observation number, on the duration of startle responses. Time taken to complete the fastest lap was used as a predictor in a further model. Random effects included in the initial model were individual specific intercepts and slopes across observations 1–8, with an assumed correlation between them. The model was first fitted using maximum likelihood estimation so that it could be compared to alternative models where (i) random intercepts and slopes were uncorrelated, and (ii) only random intercepts were included, using corrected Akaike information criterion (ΔAICc) values, where a more complex model was favoured over a simpler one if its AICc value was at least three points lower. Once the appropriate random effects structure was established we re-ran the model using restricted maximum likelihood estimation to test the fixed effects, using the Satterthwaite method to calculate degrees of freedom. Finally, we calculated (linear mixed effects model based) repeatability of startle response duration. See the electronic supplementary material, S3 for details of the code and R packages used.

## 3. Results

**Figure 1.**
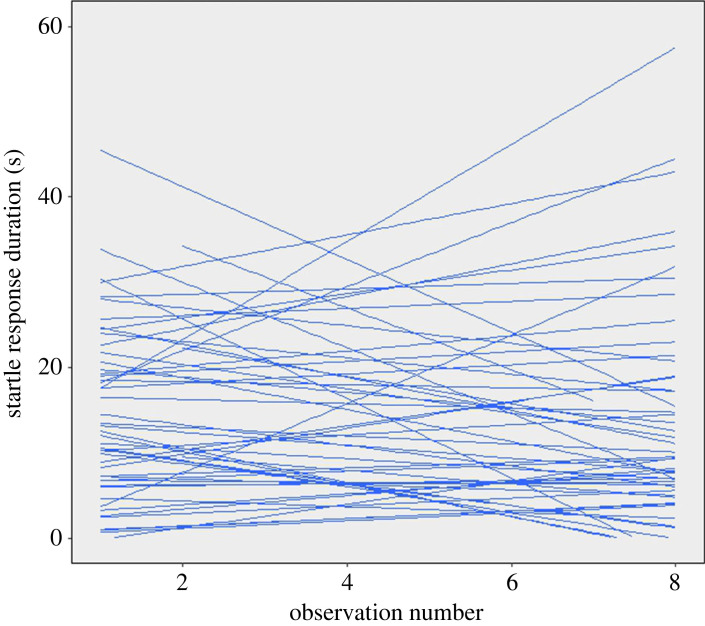
Fitted lines representing individual norms of reaction in startle response duration across repeated observations.

**Figure 2.**
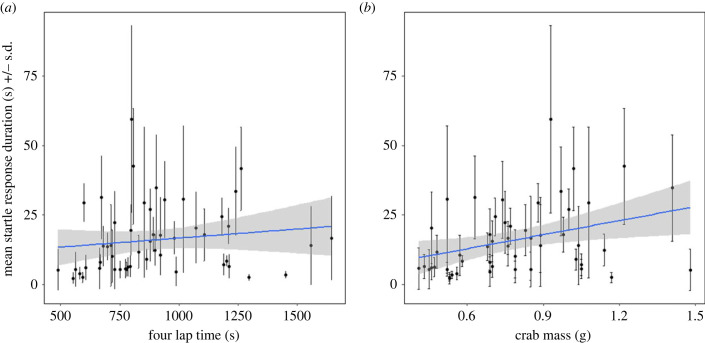
Individual means (black circles) with standard deviations (s.d.; black vertical lines) of startle response durations plotted against (*a*) time taken to walk 13 m and (*b*) crab mass. Blue lines represent ordinary least squares linear regressions of mean startle response durations against (*a*) time taken to complete four laps and (*b*) crab mass, and shaded areas show the standard error of the regression.

Individuals differed in within-individual variance (Levene's test: *F*_50,356_ = 3.2, *p* < 0.0001) but startle responses were still repeatable (*R* = 0.61 [95% confidence interval (CI) = 0.473, 0.699], *P*_LRT_ < 0.0001). The model including correlated random intercepts and slopes provided the better fit for the data compared with the model containing uncorrelated random effects (ΔAICc = 3.7) or random intercepts only (ΔAICc = 16.9), indicating significant variation among individuals in how their startle responses changed across observations (figure 1). Startle response duration did not vary across observations (*F*_1,50.04_ = 1.3, *p* = 0.26) or correlate with time to complete four laps (*F*_1,48_ = 2.85, *p* = 0.1; figure 2*a*) but the duration increased on average with crab mass (*F*_1,48_ = 5.26, *p* = 0.026; figure 2*b*). Results using time taken to complete the fastest lap were qualitatively identical and are reported in the electronic supplementary material, S2, which also outlines an alternative analytical approach.

## 4. Discussion

Startle response duration is frequently used as an index of boldness (e.g. [1,4,11–14]), and for probing motivational states (e.g. [21,22]). Here there was no association in either direction between locomotion speed and mean startle responses. Thus, it is unlikely that locomotive performance contributes to repeatable variation in startle response duration in hermit crabs, either as a constraint on dynamic performance capacity, or as an alternative strategy to hiding. In a previous study [23] we found a link between startle response duration and a potential casual factor for dynamic performance capacity, haemocyanin concentration, the respiratory pigment that determines oxygen carrying capacity. In this case, the correlation was in the opposite direction to the expectation under constraints on performance limiting the speed of re-emergence because startle response duration increased rather than decreased with haemocyanin concentration. Thus, it appeared that individuals in good physiological condition behaved in a more risk averse way rather than being more risk prone, perhaps because greater oxygen storage allowed them to tolerate a longer period of respiratory limitation while withdrawn into the shell [23], or because (as suggested above) investment in haemocyanin is part of a wider risk-mitigation syndrome.

The current null result for dynamic performance provides further evidence to support the view that startle response duration in hermit crabs provides a direct index of boldness, i.e. variation in risk-coping, albeit one where oxygen carrying capacity may contribute to an upper limit of hiding times [23]. While there was no relationship with dynamic performance, other patterns in the current analysis are consistent with previous studies of boldness in hermit crabs. Boldness was repeatable [12,20,23], it increased with crab mass [24] and individuals differed in variance around their means [2,4,25]. Individuals also differed in how their startle responses changed over repeated observation [4]. Visual inspection of individual slopes indicates that some crabs sensitized (i.e. startle response durations increased with observation number), some habituated (i.e. startle response durations decreased with observation number), but for most crabs there was limited overall change in either direction.

Similar patterns are seen in other animal personality studies focussed on boldness and using startle responses or analogous latency measures (e.g. [4]). We suggest that testing for links between such measures and dynamic performance would clarify the extent to which these patterns represent variation in boldness *per se*. The presence of a correlation where latency decreases as performance capacity increases (note that in the current experiment this would equate to a positive correlation between startle response duration and time taken to complete the locomotory task) could be owing to an indirect link between the repeatable behaviour and risk-coping. Alternatively, such a correlation could be present because the repeatable behaviour under test primarily relates to dynamic performance capacity rather than risk-coping. Thus, additional data would then be needed to determine the underpinnings of repeatable latency behaviour. One potential approach would be to collect repeated measures of dynamic performance per individual, ideally time-matched with the collection of repeated startle response data. This would enable between- and within- individual covariation to be distinguished [25] across different conditions of risk exposure (e.g. a predator cue absent and present) and energetic state (which will impact on dynamic performance). Then the relative contribution of each to the means and variances of startle response duration could be assessed. Furthermore, an interaction between risk-level and performance capacity could be tested for. For instance, in systems where dynamic performance capacities do impose constraints on recovery time, such constraints might be greater under low-risk situations where latencies are expected to be relatively short in species that show behavioural plasticity over gradients of risk. By contrast, under high-risk situations with elevated hiding times, we would not expect performance capacity to constrain latency to recover. In the current study though, we found no evidence that startle responses covary with dynamic performance capacity indicating that latency of re-emergence is independent of this measure. This allows us to be less ‘agnostic' [6] in our interpretation of repeatable startle response durations and lends more confidence to the assumption that they represent consistent differences in risk-coping strategy, or ‘boldness' in terms of how this phrase is generally understood.

## Data Availability

Data are available in the electronic supplementary material: DPSR_LM data.csv [26].
